# Comparative Evaluation of Functional Properties of Cow, Goat, and Donkey Milks Fermented with Lactic Acid Bacteria

**DOI:** 10.3390/antiox14111331

**Published:** 2025-11-05

**Authors:** Giusy Rita Caponio, Alessandro Annunziato, Mirco Vacca, Mariasimona Cavaliere, Ilenia Ceglie, Marianna Ranieri, Alessio Di Luca, Angela Gabriella D’Alessandro, Grazia Tamma, Maria De Angelis

**Affiliations:** 1 Department of Bioscience, Biotechnology and Environment, University of Bari Aldo Moro, Via Orabona 4, 70125 Bari, Italy; giusy.caponio@uniba.it (G.R.C.); marianna.ranieri@uniba.it (M.R.); grazia.tamma@uniba.it (G.T.); 2 Department of Soil, Plant and Food Sciences, University of Bari Aldo Moro, Via Amendola 165/A, 70126 Bari, Italy; alessandro.annunziato@uniba.it (A.A.); m.cavaliere14@phd.uniba.it (M.C.); i.ceglie97@gmail.com (I.C.); alessio.diluca@uniba.it (A.D.L.); angelagabriella.dalessandro@uniba.it (A.G.D.); maria.deangelis@uniba.it (M.D.A.)

**Keywords:** fermented milk, lactic acid bacteria, donkey milk, goat milk, antioxidant activity, reactive oxygen species (ROS), antimicrobial activity

## Abstract

Milk represents not only a source of essential nutrients but also a versatile matrix for the development of functional foods when combined with lactic acid bacteria (LAB) fermentation. While cow milk is the most widely consumed, alternative matrices such as goat and donkey milk possess distinctive compositional features that may influence the bioactivity of fermented products. In this work, fifteen different strains belonging to LAB and bifidobacteria were initially tested for their ability to ferment cow, goat, and donkey milk. Fermented samples showing the best acidification rate were further screened for total phenolic content (TPC), radical scavenging activity (RSA), and effects in human colon epithelial cells and Caco-2 adenocarcinoma cells. The results showed that fermentation modulated TPC in a strain- and matrix-dependent manner, with donkey milk showing the most consistent increases. RSA was significantly enhanced in fermented samples, particularly those inoculated with *Lacticaseibacillus casei* BGP93 and LC4P1 strains and *Limosilactobacillus reuteri* ATCC23272. Cell viability assays confirmed the absence of cytotoxicity, and fermented milk extracts reduced ROS under induced oxidative stress. Antimicrobial assay showed that *Lactiplantibacillus plantarum* LPAL selectively inhibited *Listeria monocytogenes*, with the strongest effect observed in donkey milk. These findings suggest that LAB-fermented milks from goats and donkeys may represent safe functional foods with improved antioxidant and antimicrobial activities.

## 1. Introduction

Milk and dairy products represent a key component of human nutrition, not only as a primary source of macronutrients such as proteins, lipids, and carbohydrates, but also as a source of bioactive compounds with health-promoting potential [1]. Beyond the fundamental role in infant feeding, dairy products continue to be consumed throughout adulthood due to their digestibility, versatility, and nutritional value. Over the past few decades, the interest in milk has extended beyond its basic nutritional role, with growing attention to its function as a vehicle for bioactive compounds, particularly when fermented [2,3]. Fermented milks have been extensively studied for their impact on gut health, immune regulation, antioxidant defense, and metabolic balance, defining them as promising candidates for the development of future functional foods [4,5,6,7].

Other than cow milk (CM), which is widely used in global dairy production, increasing attention is directed toward milks different than CM, such as goat (GM) and donkey (DM), due to their distinctive biochemical characteristics and relevance to specific consumer groups [8,9]. GM is widely appreciated for its high digestibility and lipid composition, especially for its high content of medium-chain fatty acids and for the presence of β-caseins A2 [10,11]. Instead, DM is recognized for its hypoallergenic features being closer to human breast milk and for the presence of unique bioactive components, including high lysozyme, lactoferrin, and *α*-lactalbumin contents, vitamins, and peptides [12]. Comparative analyses have underlined specific features in these milks according to their protein and fatty acid profiles, and micronutrient composition that, in turn, also affect their technological properties and health-related functions. Notably, DM has been linked to anti-inflammatory, antimicrobial, and antioxidant effects [13,14], whereas GM has shown potential benefits in cardiovascular health and lipid metabolism [15,16]. Despite these promising attributes, the use of these milks to produce fermented products remains less explored when compared with CM.

Fermentation with lactic acid bacteria (LAB) and bifidobacteria is a cornerstone of dairy biotechnology [17,18]. The group of LAB encompasses various genera that have historically been used as starter cultures due to their safety, metabolism, and capacity to modulate the organoleptic and nutritional properties of milk [19,20]. Bifidobacteria, although less applied in dairy fermentation, are recognized for their probiotic potential and ability to modulate human gut microbiota [21,22]. The metabolic activity of these microorganisms is essential to hydrolyze milk proteins into bioactive peptides, lactose/glucose into lactic acid and other metabolites, and polyphenols or other microconstituents into compounds with enhanced bioavailability and, for this reason, bioactivity. Thus, fermented dairy products are increasingly recognized as promising vehicles for delivering a wide range of health benefits [23]. Among their functional attributes, antioxidant and antimicrobial activities are usually ascribed to their consumption. Oxidative stress, defined as an imbalance between reactive oxygen species (ROS) generation and antioxidant defenses, is a key factor in the development of chronic illness, such as cardiovascular disease, neurodegeneration, diabetes, and cancer [24,25,26]. On the other hand, foods, including fermented milks, enriched with polyphenol derivatives, microbial metabolites, and bioactive peptides can counteract oxidative stress by scavenging free radicals [27,28,29,30,31]. Previous works have shown that LAB fermentation enhances the radical scavenging activity (RSA) of dairy products in a strain-dependent manner, emphasizing the importance of microbial selection to design innovative functional foods [32,33,34].

The antimicrobial potential of fermented dairy products is also of considerable interest for both food preservation and human health. LAB produce organic acids, bacteriocins, hydrogen peroxide, and other metabolites capable of inhibiting microbial pathogens, including *Listeria monocytogenes*, *Salmonella* spp., *Escherichia coli*, and various fungi, and this can provide a huge contribution in enhancing product shelf life and safety [35,36,37,38,39,40,41]. However, the antimicrobial activity can vary depending on the microbial strain, fermentation conditions, and milk substrate.

While previous evidence has widely investigated the benefits derived from fermented CM, less is known about the functional outcomes of fermenting milks different to CM, such as those from GM and DM. A limited number of studies have explored how different LAB and bifidobacterial strains can modify milk from different origins, influencing their antioxidant and antimicrobial properties. Considering the compositional differences among milks, clarifying these features is essential to identify optimal milk–microorganism combinations that can maximize functional benefits.

Therefore, the present study aimed to investigate the functional properties of fermented cow, goat, and donkey milks. By integrating biochemical assays with cell-based analyses, this work provides new insights into the functional enhancement of alternative milks through microbial fermentation and highlights the strain- and matrix-dependent nature of these bioactivities.

## 2. Materials and Methods

### 2.1. Milk Samples

The study was performed using milks from three different species, collected from farms in the Apulia region (Southern Italy). The animals were reared under a semi-extensive system (pasture supplemented with concentrate), subjected to regular milking twice a day. Cow’s milk (CM) was used as a control sample, while goat’s milk (GM) was obtained from a local Italian breed (Garganica), and donkey’s milk (DM) from a local breed (Martina Franca). Except for GM, the milks were collected as bulk milks. After collection in previously sterilized glass bottles (1 L), samples were delivered at refrigerated temperature (4 ± 2 °C) to the laboratory of the Department of Soil, Plant and Food Science (DiSSPA) at the University of Bari (Italy), where their pH was measured (CM: 6.6 ± 0.2, DM: 7.13 ± 0.2 and GM 6.48 ± 0.2) before being pasteurized (70 °C for 2 min). Thus, they were frozen until their use as fermentation substrates.

### 2.2. Selection of Cell Cultures for Milk Fermentation

Fifteen strains of *Bifidobacterium* (*B.*) and lactic acid bacteria (LAB), basonym *Lactobacillus*, belonging to the culture collection of DiSSPA were used. In detail, these were *B. animalis* 13A, *B. breve* 15A, *Lactobacillus* (*L.*) *acidophilus* LA3, *L. delbrueckii* SP5, *Lacticaseibacillus* (*Lc.*) *casei* BGP93, *Lc. casei* LC4P1, *Lc. paracasei* 14A, *Lc. rhamnosus* LRB, *Lactiplantibacillus* (*Lp.*) *plantarum* 3ON, *Lp. plantarum* 8VEG3C, *Lp. plantarum* LPAL, *Lp. plantarum* ONI3, *Lp. plantarum* VEGI1, *Lp. plantarum* VEGII1, and *Limosilactobacillus* (*Ls.*) *reuteri* ATCC23272 [42]. Cells were grown twice at 37 °C for 16–18 h in De Man, Rogosa, and Sharpe (MRS) broth (Oxoid; Basingstoke, England, UK) until the stationary phase of growth. Subsequently, cells were harvested by centrifugation (10,000× *g* for 10 min at 4 °C), washed twice with 20 mM sterile potassium phosphate buffer (pH 7.0), and used to inoculate 10 mL of sample of cow (CM), goat (GM), and donkey milk (DM) at a final cell density of 7 log_10_ CFU mL^−1^, measured by optical density (OD) at a wavelength of 620 nm [43], then verified by plate count. For the fermentation process carried out in the milks, a temperature of 30 °C was selected as a compromise condition to ensure the growth of all strains while moderating the rate of acidification due to the long fermentation time (24 h). Thereby, this provided a sufficient timeframe to evaluate metabolic activities related to polyphenol transformation across the different milk matrices. After fermentation, samples were frozen for storage before further analysis.

### 2.3. Characterization of Fermented Milks

#### 2.3.1. Preparation of Milk Extracts

Hydro-alcoholic extracts from milk samples were obtained as detailed by Gu et al. [44], with slight modifications. In detail, an aliquot (1 mL) of each sample was added to 10 mL of methanol–water solution (80:20 *v*/*v*). The mixture was stirred for 1 h at 150 rpm at 4 °C, placed in a bath of ice while undergoing sonication for 15 min (Bendelin Sonorex, Berlin, Germany), then centrifuged for 10 min at 4 °C, 10,000× *g* (mod. 5804R, Eppendorf, Hamburg, Germany) to collect the supernatant, while the pellet was discarded.

#### 2.3.2. Total Phenols Content (TPC)

The TPC of extracts was studied according to the Folin–Ciocalteu (F–C) method [45], with some modifications. The F–C reagent was used, and a standard calibration curve (R^2^ = 0.9991) was prepared using different concentrations of gallic acid (GA) in methanol (0.025–0.200 mg/mL). An aliquot (50 µL) of each extract was mixed with 50 µL of F–C reagent, 50 µL of MeOH, and 250 µL of distilled water. In addition, 200 µL of sodium carbonate (20% *w*/*v*) and 400 µL of water were added and the solution was incubated at 30 °C for 90 min. The absorbance was then measured against a blank at 700 nm (Perkin Elmer, Lambda Bio 20, Boston, MA, USA). The results were expressed as mg of GA-equivalent per gram of extract (mg GAE/g).

#### 2.3.3. Radical Scavenging Activity (RSA)

The RSA of extracts was assayed according to previously defined procedures [46], with some modifications. A calibration curve (R^2^ = 0.9934) was prepared using different concentrations of GA in methanol (0.25–2.5 g mL^−1^). Then, 350 µL of each extract were added to 650 µL of a methanolic solution 0.1 mM of 2,2-diphenyl-1-picrilyhydrazil (DPPH•) radical. The solution was then vigorously mixed and incubated at 30 °C for 30 min in the dark. The decrease in the DPPH• radical concentration, expressed as a percentage, was measured as a decrease in absorbance at 517 nm using a UV–vis spectrophotometer (Agilent Cary 60 spectrophotometer; Cernusco, Italy) against a blank.

### 2.4. Functional Evaluation of Fermented Milks

#### 2.4.1. Cell Culturing Conditions

To perform the experiments, two different human cell lines were used. Specifically, the cell lines were the Caco-2 (human colorectal adenocarcinoma) (supplied by ATCC, HTB-37™, Manassas, VA, USA) and HCEC-1CT (human colon epithelial) (supplied by Evercyte, CkHT-039-0229, Vienna, Austria) cells. Caco-2 were maintained at 37 °C, 95% air, 5% CO_2_ in DMEM supplemented with fetal bovine serum (10% *v*/*v*), non-essential amino acids (1% *v*/*v*), 100 U mL^−1^ penicillin, and 100 µg mL^−1^ streptomycin. The HCEC-1CT were grown in DMEM supplemented with 2% cosmic calf serum, 20 ng mL^−1^ EGF, 10 µg mL^−1^ Insulin, 2 µg mL^−1^ Apo-Transferrin, 5 nM Sodium-Selenit, 1 µg mL^−1^ Hydrocortisone, 100 I.U. mL^−1^ penicillin, and 100 µg mL^−1^ streptomycin at 37 °C in 5% CO_2_.

#### 2.4.2. Cell Exposure to Fermented Milk Extracts

Before utilization, the fermented milks were neutralized to pH 6.9 using 35% NaOH and centrifuged at 18,500× *g* for 20 min. To eliminate the residual turbidity, the supernatant was filtered through 0.45 µm cellulose acetate membranes and stored at −80 °C until further analysis. Cells were left under basal conditions or treated for 24 h with milk supernatants.

#### 2.4.3. Calcein-AM Cell Viability Assay

HCEC-1CT and Caco-2 cells were treated as described above. Briefly, cells were plated in 96-well plates and grown to 90% confluences. Cells were left under basal conditions (untreated, C-) or treated with milk extracts (dilution 1:4) for 24 h. To evaluate the cell viability after the exposure to fermented milk extracts, cells were incubated with calcein-AM (1 µM) at 37 °C for 45 min, and then the fluorescence signal was measured and analyzed using a fluorimeter FLUOstar Omega (5.10 R2, BMG LABTECH, Offenburg, Germany) at excitation and emission wavelengths of 508 and 529 nm, respectively.

#### 2.4.4. Reactive Oxygen Species (ROS) Detection in Cell Lines

ROS were evaluated as previously detailed [47]. After treatment with extracts from fermented milks, cells were incubated with dihydro rhodamine-123 (10 μM) at 37 °C for 30 min and then recovered into a complete medium 30 min before to carry out cell lysis in RIPA buffer containing 150 mM NaCl, 10 mM Tris-HCl pH 7.2, 0.1% SDS, 1.0% Triton X-100, 1% sodium deoxycholate, and 5 mM EDTA. After centrifugation (12,000× *g* for 10 min at 4 °C), the supernatants were used for ROS detection. The fluorescence emission signal was recorded using a fluorimeter FLUOstar Omega (BMG LABTECH, Offenburg, Germany) at excitation and emission wavelengths of 508 and 529 nm, respectively. As a positive control, cells were treated with tert-butyl hydroperoxide (tBHP, 2 mM for 30 min).

#### 2.4.5. Screening for Antimicrobial Activity

Fermented milks were also screened for their potential to exert antimicrobial activity. The evaluation was based on the agar spot method as described by Tarannum et al. [48], with slight modifications. A fresh culture of *Listeria monocytogenes* was grown twice in Brain Heart Infusion (BHI) broth (MilliporeSigma by Sigma-Aldrich; St. Louis, MO, USA) for 18 h at 37 °C and then used to inoculate 20 mL of BHI agar at a final cell density of 7 log_10_ CFU mL^−1^. The microbial suspension was used for absorbance reading at 620 nm considering cultures of 0.250 corresponding to 10^8^ CFU mL^−1^, then verified by plated counts on BHI agar. Wells with a diameter of ca. 5 mm were created using sterile pipette tips and 50 µL of each fermented milk was added to each well. Plates were left for 30 min to dry and then incubated at 37 °C for 24 h. Chloramphenicol (Sigma-Aldrich), at a final concentration of 0.01 g L^−1^, and sterile water were used as the negative and positive control, respectively. The development of a clear zone of inhibition (≥2 cm) around the spot was considered as evidence of positive inhibition.

### 2.5. Statistical Analysis

The results were expressed as the mean ± standard deviation (SD) or as the mean ± standard error of the mean (SEM). Significant differences (*p*-value; *p* ≤ 0.05) were determined using a unidirectional analysis of variance (ANOVA), followed by Tukey’s test for multiple comparisons. The statistical analysis was carried out using the statistical software GraphPad Prism 10.3.1 (Boston, MA 02110, USA) statistical software.

## 3. Results

### 3.1. Microbial Culture Selection

**Figure 1:**
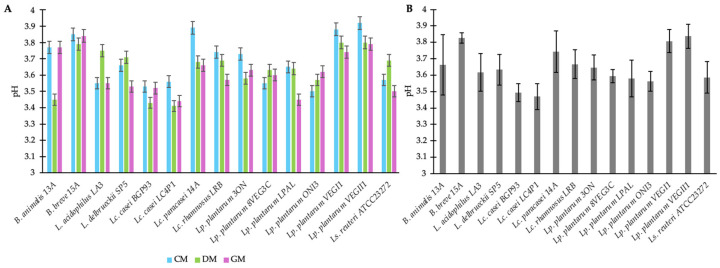
Acidification rate of fermented milks from different origins—cow (CM), donkey (DM), and goat (GM)—by strains belonging to the genera *Bifidobacterium* (*B.*), *Lactobacillus* (*L.*), *Lacticaseibacillus* (*Lc.*), *Lactiplantibacillus* (*Lp.*), and *Limosilactobacillus* (*Ls.*). Panel (**A**) shows the mean ± standard deviation (±SD) of pH values reached by individual strains fermenting each milk type (CM, DM, and GM). Panel (**B**) shows the overall mean ± SD of pH values across the three milk types.

Fifteen different cultures of bifidobacteria or LAB were screened for their capability to grow and acidify milks from different origins (cow, donkey, and goat). Great variability was observed among the strains, with LAB generally reporting a higher acidifying activity than bifidobacteria in each milk type (Figure 1A); this indicated good growth and metabolic activity in the specific food matrix. Strains reporting pH values lower than 3.6 across the three milk types (Figure 1B), supporting the moderate-to-high metabolism of organic acid despite the suboptimal conditions of growth (i.e., 30 °C), were selected for further analysis. In detail, these were *Lacticaseibacillus* (*Lc.*) *casei* BGP93, *Lc. casei* LC4P1, *Lactiplantibacillus* (*Lp.*) *plantarum* 8VEG3C, *Lp. plantarum* LPAL, *Lp. plantarum* ONI3, and *Limosilactobacillus* (*Ls.*) *reuteri* ATCC23272.

### 3.2. Total Phenol Content (TPC)

**Figure 2:**
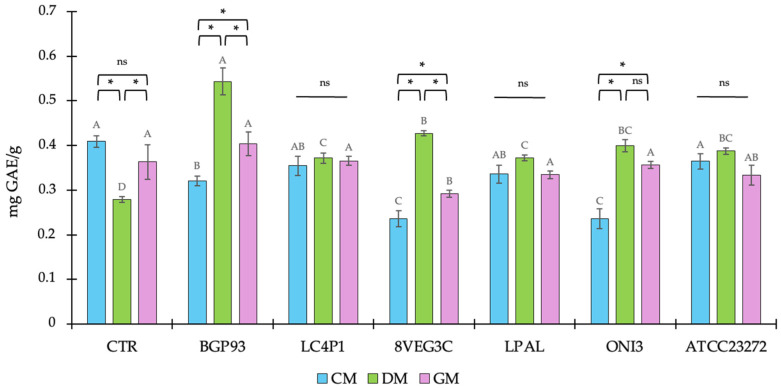
Total phenolic content (TPC) of unfermented (CTR) and fermented milks of different origins—cow (CM), donkey (DM), and goat (GM)—using various lactic acid bacteria strains. Values are expressed as mean ± SD. Asterisks (*) indicate significant (*p* < 0.05) differences between different milks subjected to the same processing, while different letters (^A–D^) denote significant (*p* < 0.05) differences among different fermentation treatments among the same milk type. In the comparison of different milks subjected to the same processing, “ns” indicates the absence of significance (*p* > 0.05).

The total phenol content (TPC) of the unfermented milks from different origins and the related samples fermented using different LAB strains are reported in Figure 2. In the unfermented milk controls (CTRs), the TPC was similar between CM (0.409 ± 0.012) and GM (0.363 ± 0.038), and both showed a significantly higher value than DM (0.279 ± 0.006). It was previously demonstrated that the different milk origins [49], as well as the different animal species/breeds [50], led to differences in milk phenol profiles. Moreover, considering the contribution of animal feeding to milk phenols [51,52] and the different dietary habits existing between cows, donkeys, and goats, both intra- and inter-species differences were expected. Instead, it is worth noting the ratio in TPC values between milks based on different origins, since this was not further confirmed after the fermentation. In fact, all fermented DM reported a significant increase in TPC, whatever the strain used as the inoculum, whereas the same was not observed in CM and GM. In three out of six fermented CMs (8VEG3C, BGP93, and ATCC23272) and in one out of six fermented GMs (8VEG3C) a significant decrease in TPC was found, while other samples were not significantly different with respect to the related unfermented (CTR) milk. Considering the equal conditions of fermentation, this discrepancy in the behavior of LAB can be related to the different compositions of milks, both in terms of macro and, particularly, of micro (polyphenols) nutrients acting as substrates of fermentation. Previous studies have reported that different animal feeding systems had an effect on the polyphenol profile in milks [53,54], and that a relationship between phenol-enriched feeds and milk fatty acid composition also exists [55]. Moreover, the effect of fermentation on phenols can be considered multifaced [56] and, more importantly, strain-dependent [34]. Based on these considerations, we observed here that, apart from the differences in the baseline value of milks, the LAB used in this study had a different behavior in milks from different origins with DM, showing the most promising results in terms of polyphenol contents after LAB-based fermentation. This trend particularly involved the combination of DM with *Lc. casei* BGP93 because these samples reported both an increase in concentration, about two-fold, and the highest TPC values, reaching values of 0.543 ± 0.03 mg GAE/g. The observed increase in TPC in fermented milks is not due to the de novo synthesis of phenols, but rather to the bacterial release of bound forms already present in the matrix. Various strains of LAB can encode different enzymes involved in the metabolism of phenols, such as esterases, decarboxylases, reductases, and glucosidases [57]. During fermentation, the starter enzymes are expected to produce enzymes and hydrolyze the complex molecule into a simple molecule. The glucosidases group, for example, is made up of enzymes largely diffused among various strains of LAB, which are involved in the breaking of the glycosidic bonds linking phenols to sugars. This enzymatic hydrolysis liberates free phenols, making them detectable and, therefore, increasing the TPC measurement [58]. Furthermore, the subsequent biotransformation of phenolic precursors present as an inactive form contributes to the TPC value increasing.

### 3.3. Radical Scavenging Activity (RSA)

**Figure 3:**
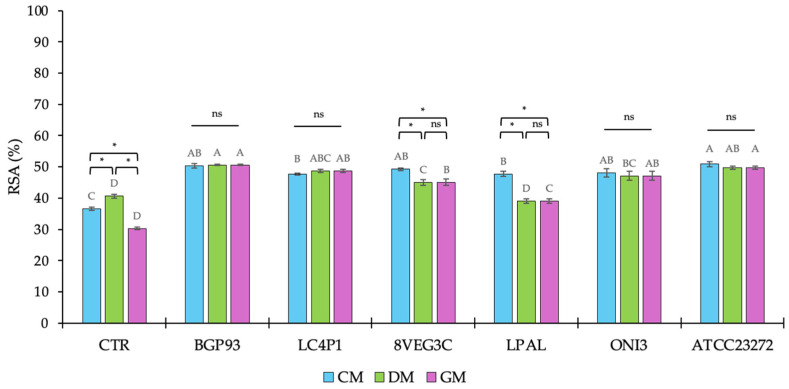
Percentage (%) of radical scavenging activity (RSA) of unfermented (CTR) and fermented milks of different origins—cow (CM), donkey (DM), and goat (GM)—using various lactic acid bacteria strains. Values are expressed as mean ± standard deviation (SD). Asterisks (*) indicate significant (*p* < 0.05) differences between different milks subjected to the same processing, while different letters (^A–D^) denote significant (*p* < 0.05) differences among different fermentation treatments among the same milk type. In the comparison of different milks subjected to the same processing, “ns” indicates the absence of significance (*p* > 0.05).

Previous studies have reported how milks from different animal species exhibiting a large variability in phenol profiles led also to dissimilarities in antioxidant activity [50,59]; as is widely known, this is mainly supported by the specific polyphenol content and composition because these micronutrients can act both as prebiotic substrates or antimicrobial agents. In this study, the antioxidant activity was determined as the RSA against the DPPH• free radical and the results are reported in Figure 3. The assay confirmed the presence of differences between unfermented milks, used as controls (CTRs), according to DM reporting the highest RSA and CM, showing a higher RSA than GM. It is noteworthy that although DM showed the lowest value of TPC, the DPPH• free radical assay demonstrated the highest percentage of RSA, suggesting that additional bioactive components of DM contributed to its overall antioxidant activity [59].

After fermentation, except for DM fermented with *Lp. plantarum* LPAL, an increase in RSA percentage was assayed in all samples regardless of the inoculum used. The moderate contribution of *Lp. plantarum* LPAL to improve RSA was also observed in GM, whereas its behavior was different in CM because, in this milk, it exhibited similar results to BGP93, LC4P1, 8VEG3, and ONI3, and was only lower than ATCC23272.

By contrast, *Lc. casei* strains BGP93 and LC4P1 yielded the highest radical scavenging activity, with values close to or slightly above 50%, independent of the milk used as the substrate, indicating that, as supported by previous studies both in vitro and in vivo [60,61,62,63,64], the antioxidant enhancement provided by *Lc. casei* strains is robust. Similarly, *Ls. reuteri* ATCC23272 showed a high antioxidant activity, close to 50% of RSA, in all fermented milks. Instead, as assessed for LPAL, another strain of *Lp. plantarum* (8VEG3C) demonstrated slight difficulties to enhance the RSA of DM and GM.

Thus, with only few exceptions, inoculating CM, DM, and GM with LAB greatly enhanced the RSA, supporting their huge contribution to obtaining functional dairy products with improved beneficial effects on human health [32,64,65,66]. Multiple components naturally present in milk, such as whey proteins, catalase enzymes, probiotics, and lactic acid, help to improve the antioxidant effect, facilitating the development of fermented products enriched in antioxidants [67,68,69,70]. Moreover, our results showed that the RSA of fermented milks is modulated by the interaction between the matrix and the starter. In fact, mechanistic studies indicate that the decrease in DPPH• free radicals does not only depend on the concentration of total phenols, because endogenous bioactive peptides of milk and those deriving from the volunteer inoculum of *Lactobacillus* and *Lactococcus* species/strains can also exert antioxidant activity [32,33,34]. This evidence further contributes to show that RSA and TPC may differ depending both on the strain used as the inoculum and the overall fermentation process conditions.

### 3.4. Functional Evaluation of Fermented Milks

#### 3.4.1. Calcein-AM Cell Viability Assay

**Figure 4:**
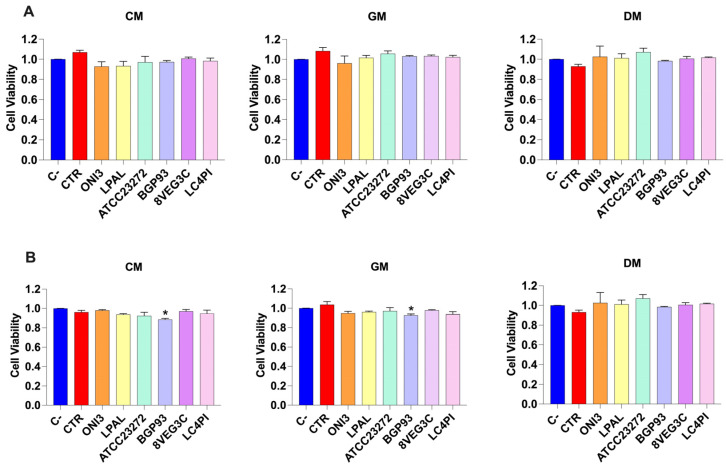
Cell viability of Caco-2 (panel (**A**)) and HCEC-1CT (panel (**B**)) cells treated with unfermented (CTR) and fermented milks of different origins—cow (CM), donkey (DM), and goat (GM)—using various lactic acid bacteria strains. Negative control (C−; vitality: 100%) is untreated cells used to evaluate the basal cell viability by calcein-AM assay. Values are expressed as mean ± standard error of the mean (SEM). Asterisks (*) indicate significant difference (*p* < 0.05) of samples compared with C− in the same milk type.

The cytotoxicity of fermented milks was tested in vitro using the Caco-2 and HCEC-1CT cellular lines. Cells were treated with milk extracts for 24 h and then the residual viability was assessed according to the calcein-AM assay. The results are reported in Figure 4. Untreated cells (C−) maintained 100% viability. None of the milk samples caused a significant decrease in cell viability compared with the C− in the Caco-2 cell line (Figure 4A). Conversely, a slight reduction in cell viability was observed in HCEC-1CT treated with BGP93 in GM and CM (Figure 4B), which may probably be due to the features of HCEC-1CT cells with respect to the fermented milk with BGP93.

#### 3.4.2. Reactive Oxygen Species (ROS) Detection

**Figure 5:**
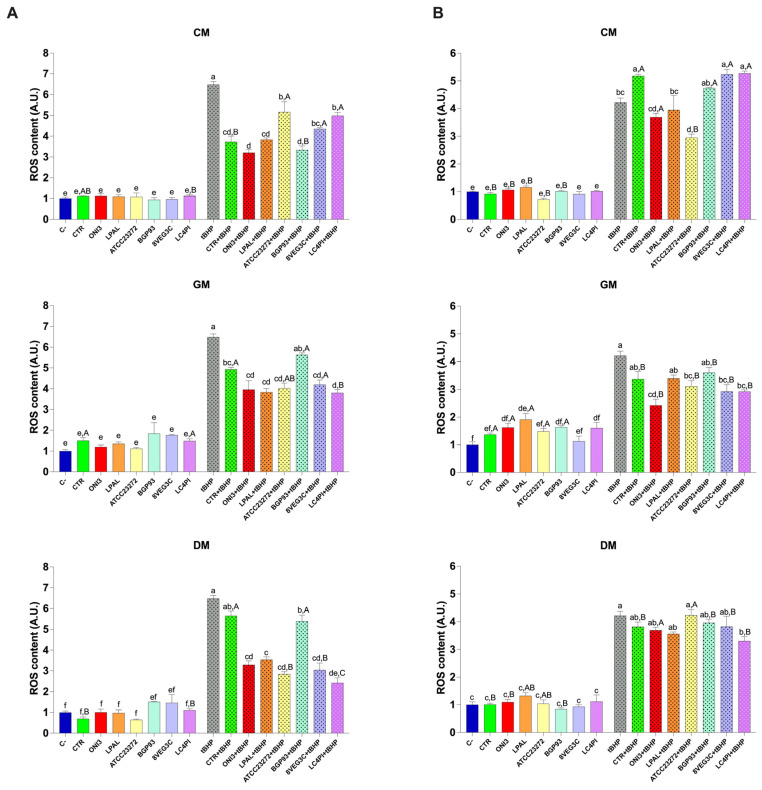
ROS content in Caco-2 (panel (**A**)) and HCEC-1CT (panel (**B**)) cells treated with unfermented (CTR) and fermented milks of different origins—cow (CM), donkey (DM), and goat (GM)—using various lactic acid bacteria strains. Values are expressed as mean ± standard error of the mean (SEM). Within the same cell line, different lowercase letters indicate significant differences (*p* < 0.05) between different treatments of the same milk type. Uppercase letters indicate significant differences (*p* < 0.05) between different milk types (CM, GM, DM) for the same treated sample.

To assess the antioxidant activity of our samples in vitro, HCEC-1CT and Caco-2 cells were treated as previously described and used to measure ROS content. As shown in Figure 5, cells treated with tert-butyl hydroperoxide (tBHP), a natural prooxidant, showed a significant increase in ROS. Treatment with CM, GM, and DM did not significantly increase ROS levels, in both cell lines, compared with the untreated control cells (C−), indicating the absence of prooxidant effects under basal conditions. Notably, when cells were exposed to treatment with milk samples in combination with tBHP, a significant reduction in ROS levels was observed. Specifically, the samples that showed the most marked antioxidant activity were GM and DM, reversing the oxidative stress induced by tBHP. Although to a lesser extent than GM, CM significantly reduced ROS levels, in particular in Caco-2 cells. Based on the milk origin, differences in ROS modulation were observed among the various inoculated strains. Overall, compared with unfermented milks (CTRs), samples fermented with LAB showed a significant reduction in ROS in GM and DM (Figure 5A). No relevant changes were detected in Caco-2 cells treated with fermented CM samples compared with CTR-CM. By contrast, in HCEC-1CT cells, significant antioxidant activity was observed in cells co-treated with tBHP and ONI3-, LPAL-, and ATCC23272-fermented milks compared with the basal milk (CTR). Together, these findings underline that differential responses are obtained in the two different cellular models of the colon. Specifically, Caco-2 cells, derived from adenocarcinoma, exhibited a stronger antioxidant response (Figure 5A), while HCEC-1CT cells (Figure 5B), which retain non-transformed epithelial features, remained more selectively responsive to specific microbial strains, as evidenced by reduced ROS levels following tBHP-induced oxidative stress.

To compare the same samples from different types of milk, we performed a further statistical analysis on cellular ROS levels. As shown in Figure 5A, under basal conditions, only the CTR and LC4PI milks significantly influenced ROS content among the three milk varieties. Specifically, DM showed a reduced ROS content for CTR and LC4PI compared with GM and, to a lesser extent, CM. However, in combination with tBHP, greater differences emerged among the samples. For instance, treatments with ATCC23272, 8VEG3C, and LC4PI caused a greater reduction in ROS in DM than in CM. Regarding the HCEC-1CT cells (Figure 5B), a similar but more distinct trend was observed. Overall, GM samples displayed the lowest ROS levels, followed by DM and CM. Specifically, treatments with ONI3, ATCC23272, and 8VEG3C showed a significantly lower ROS content in GM compared with the other two milk types (*p* < 0.05). Furthermore, LC4PI samples showed significantly reduced ROS levels in both GM and DM compared with CM, confirming the stronger antioxidant potential of these milk types in this cell line.

Consistent with previous studies investigating the bioactive effects of probiotic-fermented dairy products [4,59], the reduction in intracellular ROS following treatment with LAB-enriched milk confirms the antioxidant potential of these products. The greatest antioxidant effect was observed in GM fermented with LAB strains, which may be attributed to the unique composition of GM, which is rich in bioactive peptides and medium-chain fatty acids [71]. Interestingly, all samples enriched with LAB were able to significantly counteract tBHP-induced oxidative damage, suggesting their ability to modulate oxidative stress pathways in both intestinal and colonic epithelial cells. These results support the potential of fermented dairy products as functional foods with a protective role against oxidative damage in the intestine and, overall, provide further evidence of the increased biofunctionality of milk properly fermented with LAB supporting the derived antioxidant effect in vitro.

#### 3.4.3. Screening for Antimicrobial Activity

The unfermented (CTR) milks from different origins and the related samples fermented using different LAB strains were screened for potential antimicrobial activity; the data are reported in Table 1. The assay was validated using the positive (C+: chloramphenicol) and negative control (C−: sterile water) and showed that, among the tested LAB, only *Lp. plantarum* LPAL was able to partially (by fermenting CM and GM) or totally inhibit (by fermenting DM) the growth of *L. monocytogenes*. Thus, the antimicrobial screening highlighted clear strain- and matrix-dependent behavior among the tested LAB [35,38]. While none of the unfermented milk samples showed inhibitory activity against *L. monocytogenes*, fermentation with *Lp. plantarum LPAL* significantly enhanced the antimicrobial potential, particularly in DM, which achieved the complete inhibition of the pathogen. This effect was less pronounced in GM and CM, suggesting that the milk matrix composition plays a key role in modulating microbial metabolite production and bioactivity [36,40]. DM is characterized by a high lysozyme and lactoferrin content [13,14], which may synergize with LPAL metabolites such as organic acids or bacteriocin-like compounds [36,37,38] to enhance the antimicrobial effect. These findings corroborate previous studies reporting that *Lp. plantarum* strains can generate bioactive compounds during fermentation with broad-spectrum inhibitory effects on foodborne pathogens [32,34,64]. Therefore, the *Lp. plantarum* LPAL–DM combination emerges as a promising bio-protective system with potential applications in the development of functional and microbiologically safer dairy-like products [38].

## 4. Conclusions

This study demonstrated that the fermentation of cow, goat, and donkey milks with selected LAB enhances their functional properties in a strain- and matrix-dependent manner. DM fermented with *Lc. casei* BGP93 showed the largest increase in total phenolic content, approximately twice that of the unfermented control. The radical scavenging activity also improved in all fermented samples, with *Lc. casei* BGP93, *Lc. casei* LC4P1, and *Ls. reuteri* ATCC23272 reaching nearly 50% RSA, independent of the milk type. Cell assays confirmed that fermented milks were non-cytotoxic and reduced intracellular ROS in both Caco-2 and HCEC-1CT cells, especially in the goat and donkey milks (*p* < 0.05). The antimicrobial test identified *Lp. plantarum* LPAL-fermented DM as the only sample that completely inhibited *L. monocytogenes* growth, while only partial inhibition occurred in cow and goat milks. Taken together, these findings indicate that properly fermented donkey and goat milks exhibit enhanced antioxidant and antimicrobial activities compared with cow milk. The combinations *Lc. casei* BGP93–DM and *Lp. plantarum* LPAL–DM appear particularly promising for developing functional and microbiologically safer dairy-like products with potential use in preventive nutrition.

## Figures and Tables

**Table 1:** Evaluation of the antimicrobial effect, by diffusion assay on agar plates, of unfermented (CTR) and fermented milks of different origins—cow (CM), donkey (DM), and goat (GM)—using various lactic acid bacteria strains. Values, representing the diameter of the inhibition zone of *Listeria monocytogenes* around the well containing milk samples, are expressed as mean ± SD, with different superscript letters indicating a significant difference (*p* < 0.05; two-tailed Student’s *t*-test).

Milk	Sample	Inhibition Zone (cm)	Inhibition
	C+ ^1^	2.65 ± 0.05 ^a^	+
	C− ^2^	n.d. * ^d^	−
CM	CTR	n.d. ^d^	−
	*Lc. casei* BGP93	n.d. ^d^	−
	*Lc. casei* LC4P1	n.d. ^d^	−
	*Lp. plantarum* 8VEG3C	n.d. ^d^	−
	*Lp. plantarum* LPAL	0.7 ± 0.05 ^c^	±
	*Lp. plantarum* ONI3	n.d.	−
	*Ls. reuteri* ATCC23272	n.d.	−
DM	CTR	n.d. ^d^	−
	*Lc. casei* BGP93	n.d. ^d^	−
	*Lc. casei* LC4P1	n.d. ^d^	−
	*Lp. plantarum* 8VEG3C	n.d. ^d^	−
	*Lp. plantarum* LPAL	1.85 ± 0.05 ^b^	+
	*Lp. plantarum* ONI3	n.d. ^d^	−
	*Ls. reuteri* ATCC23272	n.d. ^d^	−
GM	CTR	n.d. ^d^	−
	*Lc. casei* BGP93	n.d. ^d^	−
	*Lc. casei* LC4P1	n.d. ^d^	−
	*Lp. plantarum* 8VEG3C	n.d. ^d^	−
	*Lp. plantarum* LPAL	0.65 ± 0.05 ^c^	±
	*Lp. plantarum* ONI3	n.d. ^d^	−
	*Ls. reuteri* ATCC23272	n.d. ^d^	−

^1^ Positive control: chloramphenicol. ^2^ Negative control: sterile water. * n.d.: no inhibition around the well (5 mm).

## Data Availability

The raw data supporting the conclusions of this article will be made available by the authors on request.

## Abbreviations

The following abbreviations are used in this manuscript:

CM	Cow Milk
GM	Goat Milk
DM	Donkey Milk
LAB	Lactic Acid Bacteria
*B.*	*Bifidobacterium*
*L.*	*Lactobacillus*
*Lc.*	*Lacticaseibacillus*
*Lp.*	*Lactiplantibacillus*
*Ls.*	*Limosilactobacillus*
OD	Optical Density
MRS	de Man, Rogosa, and Sharpe (broth/agar)
TPC	Total Phenolic Content
GA	Gallic Acid
GAE	Gallic Acid Equivalents
RSA	Radical Scavenging Activity
DPPH•	2,2-diphenyl-1-picrylhydrazyl radical
UV-vis	Ultraviolet
HCEC-1CT	Human Colon Epithelial Cell line
ROS	Reactive Oxygen Species
RIPA	Radioimmunoprecipitation Assay (buffer)
tBHP	Tert-Butyl Hydroperoxide
BHI	Brain Heart Infusion (broth/agar)
SD	Standard Deviation
SEM	Standard Error of the Mean
